# Crystal structure of the catalytic domain of botulinum neurotoxin subtype A3

**DOI:** 10.1016/j.jbc.2021.100684

**Published:** 2021-04-21

**Authors:** Oneda Leka, Yufan Wu, Xiaodan Li, Richard A. Kammerer

**Affiliations:** The Laboratory of Biomolecular Research, Division of Biology and Chemistry, Paul Scherrer Institute, Villigen PSI, Switzerland

**Keywords:** botulinum neurotoxin, catalytic domain, circular dichroism, SNAP-25, subtype A3, X-ray crystallography, BoNT, botulinum neurotoxin, CD, circular dichroism, HC, heavy chain, H_C_, receptor-binding domain, H_N_, translocation domain, LC, light chain (catalytic domain), LPH, region of low primary amino acid homology, SNAP-25, synaptosomal-associated protein 25, SNARE, soluble N-ethylmaleimide-sensitive-factor attachment receptor, SV, synaptic vesicle, SV2, synaptic vesicle glycoprotein 2, Syt, synaptotagmin, Trx, thioredoxin, VAMP, vesicle-associated membrane protein

## Abstract

Botulinum neurotoxins (BoNTs) are among the most widely used therapeutic proteins; however, only two subtypes within the seven serotypes, BoNT/A1 and BoNT/B1, are currently used for medical and cosmetic applications. Distinct catalytic properties, substrate specificities, and duration of enzymatic activities potentially make other subtypes very attractive candidates to outperform conventional BoNTs in particular therapeutic applications. For example, BoNT/A3 has a significantly shorter duration of action than other BoNT/A subtypes. Notably, BoNT/A3 is the subtype with the least conserved catalytic domain among BoNT/A subtypes. This suggests that the sequence differences, many of which concern the α-exosite, contribute to the observed functional differences in toxin persistence by affecting the binding of the substrate SNAP-25 and/or the stability of the catalytic domain fold. To identify the molecular determinants accounting for the differences in the persistence observed for BoNT/A subtypes, we determined the crystal structure of the catalytic domain of BoNT/A3 (LC/A3). The structure of LC/A3 was found to be very similar to that of LC/A1, suggesting that the overall mode of SNAP-25 binding is common between these two proteins. However, circular dichroism (CD) thermal unfolding experiments demonstrated that LC/A3 is significantly less stable than LC/A1, implying that this might contribute to the reduced toxin persistence of BoNT/A3. These findings could be of interest in developing next-generation therapeutic toxins.

Botulinum neurotoxins (BoNTs), secreted by neurotoxigenic Gram-positive strains of *Clostridia*, are the most toxic bacterial proteins known (1). BoNTs are the causative agents of botulism, a rare but severe neuroparalytic syndrome that is the pathological result of the toxins’ action on nerve terminals of both the skeletal and the autonomous nerve system (2). Classical BoNTs are currently divided into seven serotypes (BoNT/A,/B,/C,/D,/E,/F, and/G). However, their genetic variability is much larger, which mainly results from the existence of subtypes within certain serotypes (3). Despite their toxicity, BoNT/A1 and BoNT/B1 are routinely used in a steadily increasing number of cosmetic and medical applications (4, 5).

Each BoNT is synthesized as a large precursor that is cleaved into two polypeptide chains that remain connected through an interchain disulfide bond: a heavy chain (HC, ∼100 kDa) and a light chain (LC, ∼50 kDa). The HC comprises the translocation domain (H_N_) and the receptor-binding domain (H_C_) (6). Most BoNTs bind a polysialoganglioside and a protein receptor (synaptotagmin (Syt) or synaptic vesicle glycoprotein 2 (SV2)) (7). Upon receptor-mediated endocytosis into synaptic vesicles (SVs), the acidic environment within SVs triggers a conformational change in H_N_ that leads to the insertion of H_N_ into the membrane and the formation of a transmembrane channel (8). By a not yet well-understood mechanism, the LC is then translocated through the transmembrane channel into the cytoplasm.

The LC domain is a zinc-dependent protease that cleaves components of the soluble N-ethylmaleimide-sensitive-factor attachment receptor (SNARE) family of proteins, a process that blocks acetylcholine release at the neuromuscular junction and thereby causes flaccid paralysis of muscles (9). BoNT/A and E cleave synaptosomal-associated protein 25 (SNAP-25) and BoNTs B, D, F, and G cut vesicle-associated membrane protein (VAMP). Only BoNT/C cleaves two substrates, SNAP-25 and syntaxin (10).

The genetic variability of BoNTs is further increased by the identification of different BoNT subtypes within serotypes A, B, E and F, the existence of mosaic toxins, and the discovery of several BoNT-like molecules, some of which potentially represent new BoNTs (11, 12, 13, 14). Despite the diversity of BoNTs, to date, all cosmetic and clinical applications are limited to the use BoNT/A1 and to a lesser extent BoNT/B1. Although additional serotypes are currently being investigated for potential applications as pharmaceuticals, it appears important to also characterize in detail their subtypes because they might outperform conventional BoNTs with respect to biological activities such as potency, onset and duration of action, and substrate selectivity (5).

This is well documented for the most widely used therapeutic toxin BoNT/A, for which eight subtypes, termed BoNT/A1–A8, have been reported (3). They share between 84% and 97% sequence identity, and there exists experimental evidence that such low levels of sequence variations among BoNT/A subtypes could have a significant impact on *in vivo* efficacy and pharmacological applications (15, 16, 17, 18).

One well-studied example is the persistence of BoNT/A subtypes. A long persistence of up to 6 months in patients represents one of the benefits of using BoNT/A. Pellett and co-workers assessed the persistence of the BoNT/A subtypes 1–5 in primary rat spinal neurons (19). Notably, the duration of intracellular enzymatic activity of BoNT/A1, A2, A4, and A5 was shown to be at least 10 months. In contrast, the duration of the enzymatic activity of BoNT/A3 was significantly shorter and lasted for up to about 5 months (19). The duration of BoNT action appears to be an LC function, a hypothesis that is supported by experiments with GFP-LC fusion proteins that showed differences in subcellular distribution: GFP-LC/A1 localized to the host cell membrane, while GFP-LC/A3 was found in the cytosol (20). Consistent with this observation, BoNT/E1, another toxin with a shorter duration of action when compared with BoNT/A1, also localizes to the cytosol (21). Notably, BoNT/A3 is the subtype with the least conserved LC among subtypes, suggesting that these sequence differences, many of which concern residues of the α-exosite, account for the observed functional differences in toxin persistence by affecting SNAP-25 substrate binding and/or the stability of the catalytic domain fold.

The molecular mechanisms underlying the short duration of action of BoNT/A3 are largely unknown, but detailed knowledge of these mechanisms is important for developing new generations of therapeutic toxins and for improving current toxin variants and expand their pharmacological properties. Because subtype BoNT/A3 is potentially a very attractive protein for applications in therapeutic areas, where shorter duration of action than that of BoNT/A1 is required, we decided to investigate the impact of the amino acid differences on the catalytic domain fold by determining its crystal structure by X-ray crystallography, performing activity assays, and assessing LC stability by CD spectroscopy.

## Results and discussion

### Crystal structure of LC/A3

Because attempts to crystallize variants of wild-type BoNT/A3-LC were not successful, we used an inactive truncated version of the catalytic domain for structure determination. Catalytically inactive recombinant LC/A3 spanning residues Pro2–Lys417 and containing the double mutation Glu224Gln/Phe336Tyr was produced with an N-terminal cleavable 6xHis tag by bacterial expression (for details, see experimental procedures). Crystals suitable for the determination of the high-resolution structure of LC/A3 grew within 1–2 weeks, diffracted to 2.0 Å resolution and belong to the space group *P2*_*1*_*2*_*1*_*2*_*1*_, with two molecules per asymmetric unit (Table 1). The X-ray structure was determined by molecular replacement using inactive LC/A1 (PBD 1XTG) as a search model (22).

**Table 1:** Crystallographic statistics of the LC/A3 X-ray structure

	Inactive LC/A3 (Pro2-Lys417)
Data collection	
Resolution range (Å)	48.71–2.01 (2.078–2.01)
Space group	*P*2_1_2_1_2_1_
Polypeptide chains/AU	2
Unit cell parameters	
a, b, c (Å)	66.25, 97.42, 141.25
α, β, γ (°)	90, 90, 90
Observed reflections	662847 (65,749)
Unique reflections	61,562 (6025)
Multiplicity	10.8 (10.9)
Completeness (%)	96.61 (98.58)
R-merge	0.1431 (1.047)
Mean I/sigma(I)	13.26 (1.99)
CC(1/2)	0.998 (0.675)
Wilson B-factor	24.15
Refinement	
Resolution range (Å)	48.71–2.01 (2.078–2.01)
*R*_work_ (%)	20.49
*R*_free_ (%)	23.24
Protein atoms	6554
rmsd of bond lengths	0.003
rmsd of bond angles	0.639
Average *B*-factor (Å^2^)	34.46
Ramachandran plot (%)	
Favored	97.64
Allowed	2.36
Outliers	0
Crystallization condition	0.1 M HEPES pH 7.5
	10 % w/v PEG 3350
	0.2 M Proline

Values in parenthesis correspond to the high-resolution shell.

**Figure 1:**
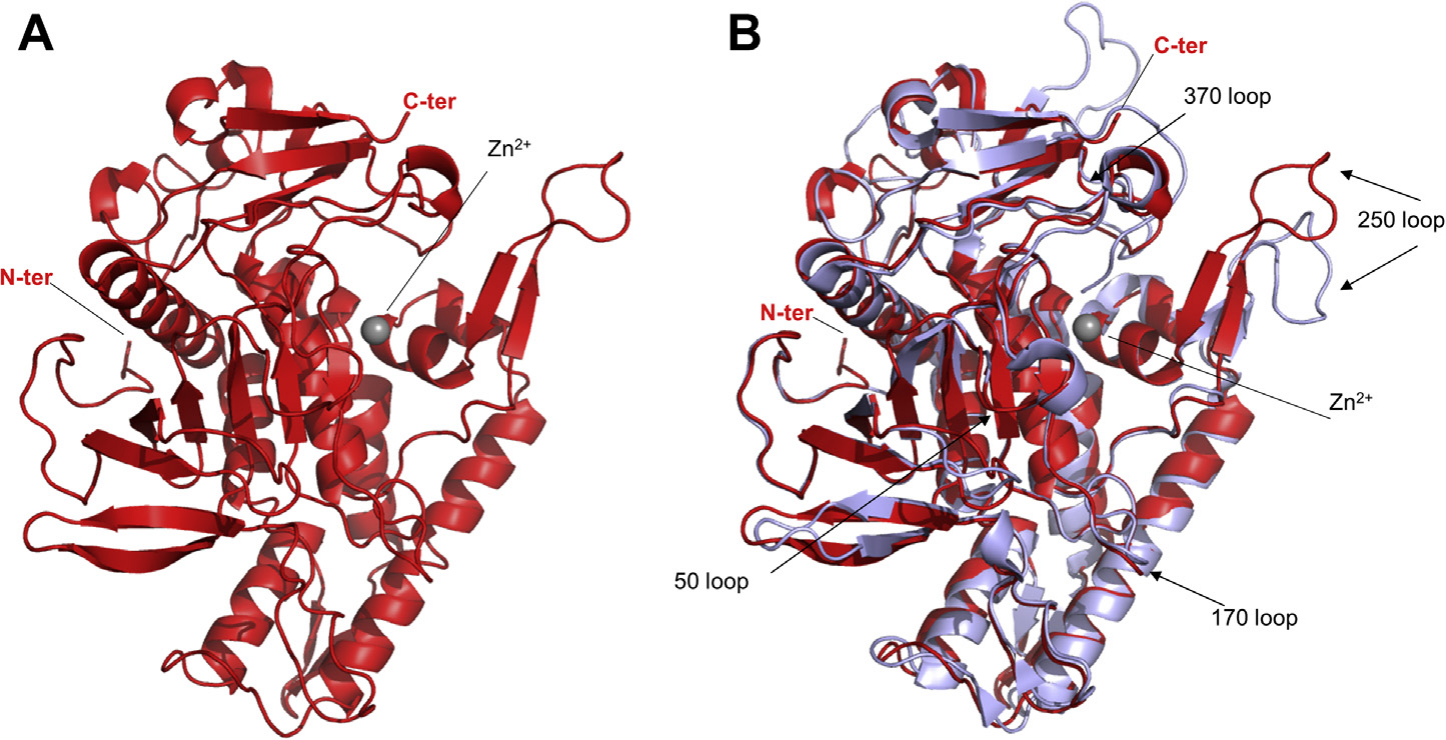
**Crystal structure of LC/A3.***A*, ribbon representation of LC/A3 (*red*, PDB code 7DVL) with the zinc ion shown as a *gray sphere*. N- and C-termini are labeled. *B*, Superimposition of LC/A3 (*red*) with LC/A1 (*magenta*, PDB code 1XTF (22); rmsd value of 0.6 Å for 343 Cα atoms of both A chains). N- and C-termini, loop regions, and the zinc ion are labeled.

The LC/A3 structure represents the typical globular BoNT-LC domain fold with a conserved groove that extends from the catalytic site around the enzyme (Figs. 1, *A* and 3, *B*). The electron density is well defined for all residues, with the exception of loop 200 (amino acid residues Ser201-Ala212 and Glu203-Ala203 of chain A and B, respectively), which is likely to be disordered. Zoom-in views of the refined electron density map generated from the final calculated phases are shown in Fig. S1.

**Figure 2:**
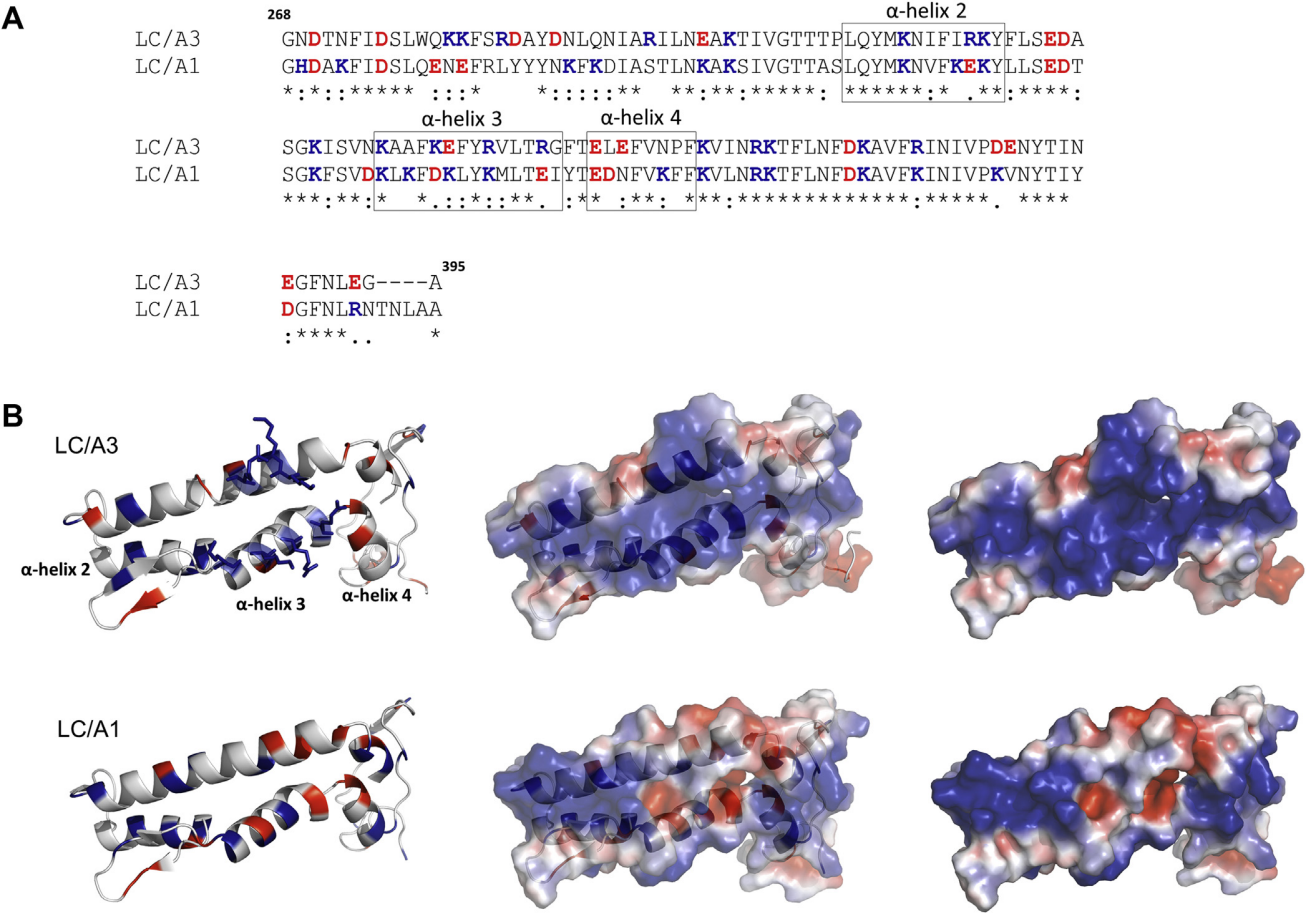
**Comparison of the low primary homology region (LPH) of LC/A3 with the corresponding amino acid sequence of LC/A1.***A*, Clustal Omega alignment (32) of the LPH region of LC/A3 and the corresponding amino acid sequence of LC/A1. The region spans residues Gly268–Ala395 of LC/A3 (20) and Gly268–Ala399 of LC/A1. α-Exosite α-helices are highlighted by boxes and labeled. ∗, identical residue, strong similarity, weak similarity. Acidic and basic residues are shown in *red* and *blue*, respectively. *B*, ribbon and surface representations of the regions shown in the alignment in (*A*). *Upper* views, ribbon representation (*left*), combined ribbon and surface representation (*middle*), and surface representation (*right*) of the LC/A3 LPH region, which contains a cluster of basic amino acids (shown as *sticks*) formed by Lys280–Arg284 and Lys335–Arg347 that is absent in LC/A1. *Bottom* view, the same representations of the corresponding amino acid sequence of LC/A1 are shown. Surface representations were colored according to their electrostatic potential (±3 kT/e, where k is the Boltzmann constant, T is temperature, and e is the elementary charge): *blue* denotes basic residues and *red* denotes acidic residues. α-Exosite α-helices are labeled.

BoNT/A3 is the subtype with the least conserved LC among subtypes and shares 81.9% sequence identity with LC/A1 (Fig. 2*S*). Sequence differences are particularly present in the amino acid segment spanning residues Gly268 to Gly395, which has been termed region of low primary amino acid homology (LPH) of LC/A3 (Fig. 2) (20). This region shares 58.8% identity with the corresponding region in LC/A1 and therefore is predicted to have a similar overall structure. Accordingly, the structure of inactive LC/A3 is virtually identical to that of the double-mutant apo structure of LC/A1 (PDB 1XTF, rmsd value of 0.6 Å for 343 Cα atoms of both A chains) and only shows minor differences in loop region 250. Loop 250 undergoes a small shift in LC/A3 when compared with LC/A1. Loops 50, 170, and 370 forming the boundaries of the large cleft on the enzyme surface are preserved in LC/A3 and LC/A1 (Fig. 1*B*).

Our structure also confirms the observation of Pellett *et al.* that the LPH region in LC/A3 contains a cluster of basic amino acids that is absent in LC/A1 (20). The cluster of basic amino acids is formed by amino acid residues Lys280 to Arg284 and Lys335 to Arg247 (Fig. 2). The overall structural organization of the LPH region, however, is conserved between LC/A1 and LC/A3 (Fig. 2*B*). In their paper, the authors reported a different intracellular distribution of LC/A1 and LC/A3 (20). Whereas LC/A1 localized to the plasma membrane of neuronal cells, LC/A3 is mainly found in the cytosol. Whether the basic cluster of amino acid residues within the LPH region determines subcellular distribution and whether the different localization of BoNT/A subtypes specifies their duration of action remains to be elucidated.

### Comparison to LC/A1-SNAP-25 structure

**Figure 3:**
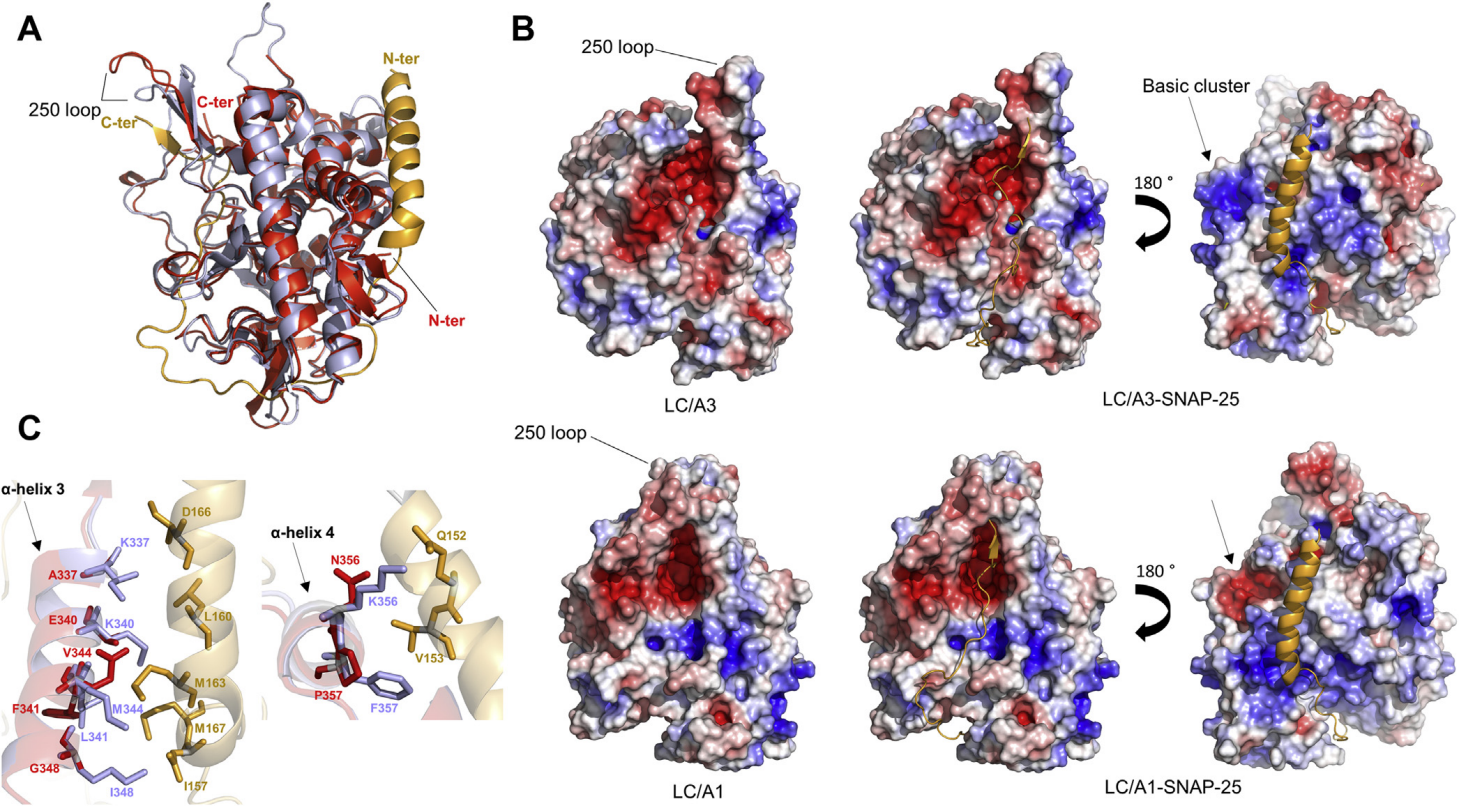
**Comparison of the LC/A3 crystal structure with the LC/A1-SNAP-25 complex.***A*, superimposed ribbon representation of LC/A3 (*red*, PDB code 7DVL) and LC/A1 (*magenta*) bound to the SNAP-25 substrate peptide (gold, PDB code 1XTG (22); rmsd value of 0.5 Å for 338 Cα atoms of both A chains). Loop 250 has an open orientation in LC/A3 and a closed conformation in the LC/A1-SNAP-25 structure. N- and C-termini of polypeptide chains are indicated. *B*, upper views, surface representation of LC/A3 (*left*) and representations of the model of the LC/A3-SNAP-25 complex showing substrate binding to the active site/β-exosite (*middle*) and the α-exosite (*right*). *Bottom* view, corresponding representations of LC/A1 and the LC/A1-SNAP-25 peptide complex. The cluster of basic amino acids (basic cluster) of the LC/A3 LPH region and the corresponding region in LC/A1 are indicated by *arrows*. Surface representations were colored according to their electrostatic potential (±3 kT/e, where k is the Boltzmann constant, T is temperature, and e is the elementary charge): *blue* denotes basic residues and *red* denotes acidic residues. *C*, superimposition of ribbon representations of LC/A1 α-exosite α-helices 3 and 4 (*magenta*) interacting with SNAP-25 (*gold*) and the corresponding helices of LC/A3 (*red*). Interacting residues are shown as sticks. α-Exosite α-helices are labeled.

The crystal structure of a SNAP-25 peptide bound to LC/A1 revealed the molecular details of substrate binding and revealed an array of exosites that are responsible for substrate-binding specificity. The α-exosite consists of four α-helices (α-helix 1, residues Asp102-Arg113; α-helix 2, residues, Leu310-Tyr321; α-helix 3, residues Lys335-Ile348 and α-helix 4, residues Glu351-Phe358) that bind a helical segment of SNAP-25 that is approximately 30–50 amino acids away from the substrate cleavage site (22). Notably, three of the four α-helices of the α-exosite (α-helices 2, 3, and 4) are found in the LPH region (Fig. 2*A*), suggesting that sequence differences might contribute to the observed functional differences in toxin persistence by affecting SNAP-25 substrate binding. As expected from the comparison with apo LC/A1, the overall structure of LC/A3 is also very similar to LC/A1 complexed with the SNAP-25 peptide (rmsd value of 0.5 Å for 338 Cα atoms of both A chains). The most pronounced difference revealed by the comparison is again observed for loop 250 that in the LC/A1-SNAP-25 complex interacts with and folds over loop 370 upon binding of the substrate at the β-exosite (Fig. 3, *A* and *B*). In our structure loop 250 has a different conformation and points away from the active site. This different orientation of loop 250 may explain the inability of LC/A3 to undergo autocleavage (23). Autocleavage has been reported for LC/A1, where a di-tyrosine (Tyr250-Tyr251) of loop 250 is cleaved by an active trans-interacting LC/A1 (24). Loop 370 is identical in both structures.

The comparison of LC/A3 with the LC/A1-SNAP-25 complex shows that both the α- and β-exosites are conserved, however, with some differences in the substrate-interacting residues (Table S1). The most significant differences of potential interactions with SNAP-25 are observed for α-exosite α-helices 3 and 4 that share 41.7% identical residues (Fig. 3*C*). Although amino acids of α-exosite α-helix 3 form part of the basic cluster of residues of the LPH (Figs. 2 and 3*B*), they are not involved in SNAP-25 binding (Fig. 3*C*, Table S1). In α-helix 3, positions 337, 340, and 348 are occupied by Lys, Lys and Ile in LC/A1 and by Ala, Glu, and Gly in LC/A3 and in α-helix 4, Lys356 and Phe357 of LC/A1 are substituted by Asn and Pro, respectively, in LC/A3. The different net charge of residues in α-exosite α-helices 3 and 4 that interact with SNAP-25, as well as the smaller size of most of these amino acids in LC/A3 when compared with LC/A1, indicates potential differences of substrate binding. However, the observation that their catalytic pockets are virtually identical and other substrate-binding residues are largely conserved (Table S1, Fig. S3) suggests that LC/A3 may accommodate SNAP-25 in a similar way as LC/A1 (Fig. 3*B*).

### *In vitro* activity of LC/A3

**Figure 4:**
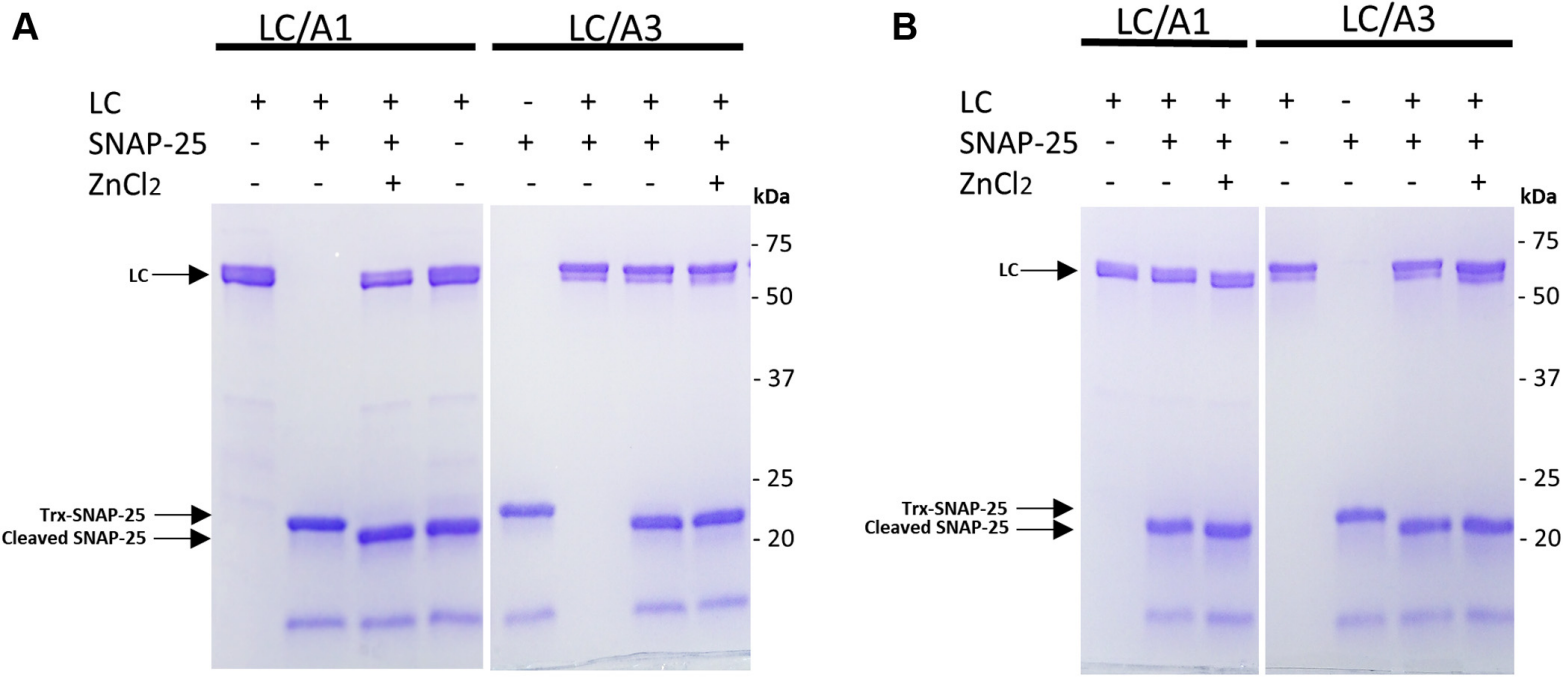
***In vitro* enzymatic activity of LC/A1 and LC/A3.***A*, 1 μg of active full-length LC/A1 (Pro2–Lys444) or LC/A3 (Pro2–Lys448) was incubated with 1 μg of Trx-SNAP-25 in 50 μl TBS buffer for 3 h at 37 °C. 10 mM ZnCl_2_ was added to the reaction mixtures, to assess whether it affects the activity of the LC/As. *B*, the same enzyme activity assay was also performed at 25 °C. Reactions were stopped by adding SDS-PAGE buffer and heated for 5 min. Samples were subjected to 4–20% gradient SDS-PAGE and gels were stained with Coomassie *Blue*. LC/A1, LC/A3, and cleaved and uncleaved substrate are indicated by *arrows*. The positions of marker proteins are indicated.

To support our structure-based conclusion that LC/A3 binds SNAP-25 in a similar manner than LC/A1, we performed substrate-cleavage assays (Fig. 4). In these experiments, we compared the cleavage of the cytosolic domain of human SNAP-25 (amino acid residues Gly146–Gly204) that is commonly used for this assay, by active full-length LC/A1 (amino acid residues Pro2-Lys448) and LC/A3 (amino acid residues Pro2-Lys444). The enzymatic reaction was performed at two different temperatures, 25 °C and 37 °C. Active full-length LC/A1 and LC/A3 completely cleaved the SNAP-25 substrate peptide at both temperatures, with or without ZnCl_2_ added to the reaction buffer (Fig. 4). These findings are consistent with a previous publication in which recombinant LC/A3 was biochemically characterized and compared with other BoNT/A subtypes (25). LC/A3 cleaved the SNAP-25 peptide substrate at half the rate and with a similar K(m) value to LC/A1.

It has been shown that full-length LC/A1 has a higher activity than truncated LC/A1 variants (26). Our results demonstrate that this is also true for LC/A3, where the truncated catalytic domain (amino acid residues Pro2-Gly417) that was initially used for crystallization trials was not able to fully cleave the SNAP-25 peptide substrate at 25 °C nor 37 °C (Fig. S4). Moreover, the stability of recombinant variants of LC/A3 seems to critically depend on the C-terminus. Truncated LC/A3 tended to degrade and precipitate at 37 °C but not at 25 °C, whereas full-length LC/A3 was stable at both temperatures.

### LC/A3 is less stable than LC/A1

**Figure 5:**
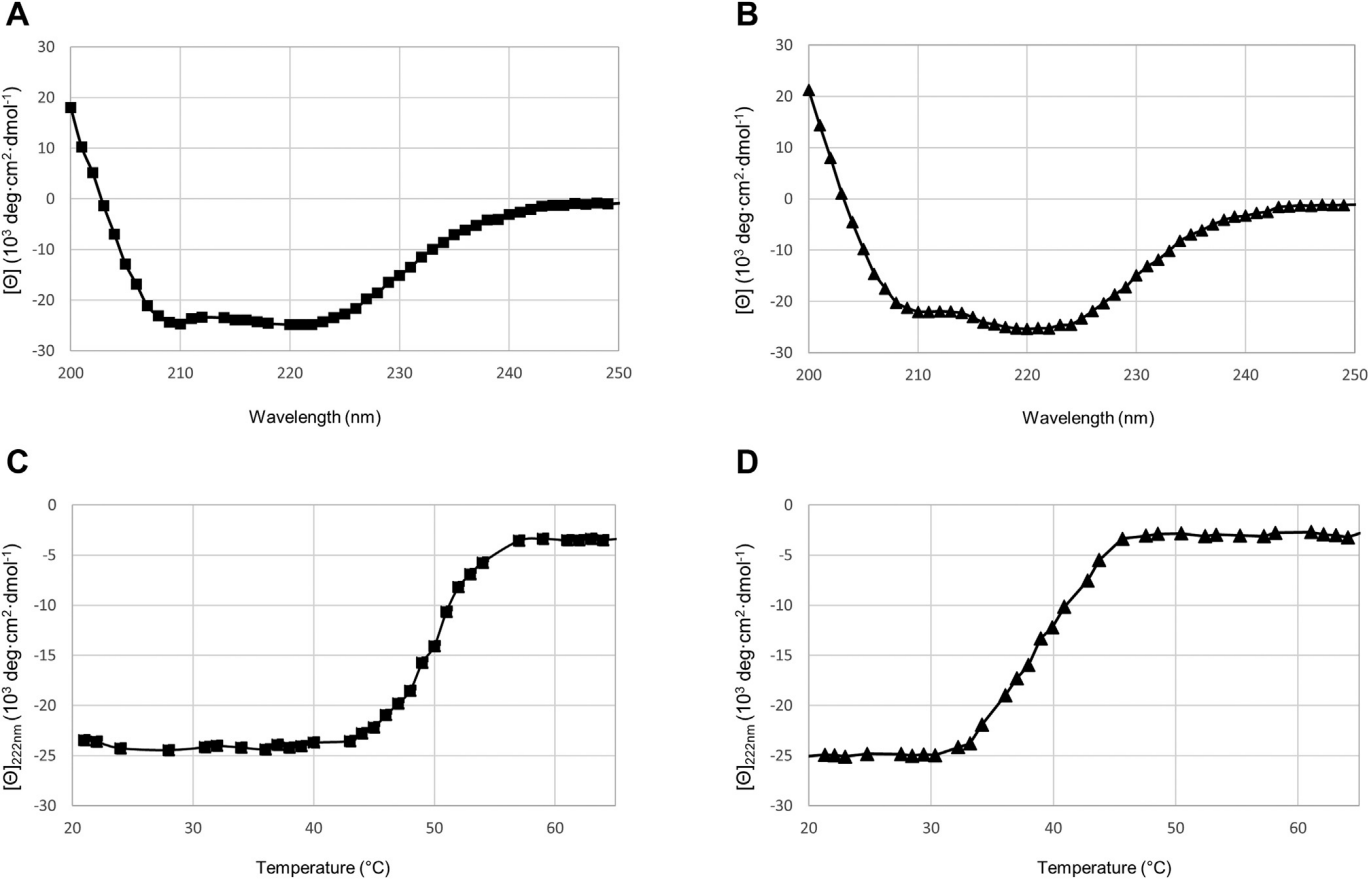
**CD spectroscopy analysis of active full-length LC/A1 and LC/A3.***A* and *B*, circular dichroism spectra of both active LC/A1 (Pro2-Lys444) and LC/A3 (Pro2-Lys448) in solution, respectively (▪, LC/A1; ▲, LC/A3). *C* and *D*, thermal unfolding profiles of LC/A1 and LC/A3, respectively, recorded at 222 nm. LC/A1 and LC/A3 showed concentration-independent T_m_ values of 51 ± 0.2 °C and 37.9 ± 0.2 °C, respectively, values that indicate that the thermal stability of LC/A3 are significantly lower than that of LC/A1. Proteins were measured at a concentration of 5 μM in PBS. Each spectrum is the average of three independent measurements. For the thermal unfolding profiles, a ramping rate of 1 °C/min was used.

CD spectroscopy was used to assess the thermal stability of active full-length LC/A1 and LC/A3. Consistent with the crystal structures of the shorter variants, the far-ultraviolet CD spectrum recorded from active full-length LC/A1 and LC/A3 showed a substantial amount of α-helicity at 20 °C with the characteristic minima near 208 and 220 nm (Fig. 5, *A* and *B*). The temperature-induced CD denaturation profile recorded from LC/A1 at 222 nm exhibited the sigmoid shape typical for a two-state transition while the thermal unfolding profile of LC/A3 was more linear (Fig. 5, *C* and *D*). We used the profiles to estimate the T_m_ of the catalytic domains. LC/A1 and LC/A3 showed concentration-independent T_m_ values of 51 ± 0.2 °C and 37.9 ± 0.2 °C, respectively, indicating that the thermal stability of LC/A3 is significantly lower than that of LC/A1. The CD experiment therefore supports our hypothesis that a lower stability of LC/A3 might contribute to the differences in the duration of action between the two subtypes, despite the crystal structures of the two domains being very similar.

Notably, the truncated variant of LC/A3 (residues Pro2-Gly417) showed a T_m_ of 40.1 ± 0.1 °C that was even slightly higher than that of the full-length protein (Fig. S5). This result is consistent with a recent report, in which the role of the C-terminus in the biological function of BoNT/A1 was investigated. Feltrup *et al*. (27) showed by several biophysical techniques that the structure of full-length LC/A1 (Pro2-Lys448) is significantly more flexible in solution than that of truncated variant (Pro2-Glu424). Furthermore, they observed stronger and faster binding of full-length LC/A1 to SNAP-25 compared with the truncated variant, which may be the reason for the dramatically higher enzymatic activity of full-length LC/A1. Therefore, the C-terminus of LC/A1 plays a critical role in introducing flexibility, which seems to be important for its enzymatic activity. The findings are also consistent with the observation that truncated LC/A fragments are easier to crystallize than the full-length catalytic domain (22). However, in contrast to these findings, the T_m_ of the truncated LC/A1 construct used in our study was lower by almost 5 °C than the full-length protein (Fig. S5). Possibly, the different results may be explained by the fact that slightly different truncated LC/A1 variants were used in the two studies.

## Conclusions

There exists strong evidence that the duration of BoNT action is primarily a property of the LC. This raises the question of which determinants of LC specify the different duration of action of BoNT/A1 and BoNT/A3. In this study, we show by X-ray crystallography that despite primary sequence differences observed between the two subtypes, their crystal structures are virtually identical. Furthermore, a comparison with the crystal structure of LC/A1 in complex with a SNAP-25 peptide indicates that the mode of substrate binding is likely to be similar in BoNT/A1 and BoNT/A3. This conclusion is supported by a previous study comparing the enzymatic activities of BoNT/A subtypes 1–4 (24) as well as our functional assays that demonstrated complete cleavage of the substrate by full-length LC/A1 and LC/A3. However, we found that one significant difference between LC/A1 and LC/A3 is their thermal stability. LC/A3 has a T_m_ value that is close to physiological body temperature, suggesting that a lower stability of the catalytic domain might contribute to the shorter persistence observed for this subtype that might be more susceptible to proteases. Experiments with LCs from BoNT/E and F, two serotypes with a shorter duration of action than BoNT/A1, as well as engineered LC/A1 and LC/A3 variants with different stabilities, might clarify if stability is an important factor of persistence. Such findings therefore could have a significant impact on the design of BoNT variants with different durations of action. Such variants are of particular interest for medical applications where different durations of action when compared with BoNT/A1 would be beneficial.

## Experimental procedures

### Protein expression and purification

Codon-optimized synthetic DNA fragments encoding inactive and active LC/A3 variants (UniProtKB entry D3IV24) spanning amino acids Pro2–Gly417 and Pro2–Lys444 were were cloned into the BamHI/EcoRI site of variants of the expression vectors pET-15b and pET-20b, respectively. Codon-optimized synthetic DNA fragments of active LC/A1 variants (UniProtKB entry P0DPI1) spanning residues Pro2-Gly421 and Pro2-Lys448 were cloned as described for the LC/A3 variants. pET-15b was modified to contain N-terminal MKKHHHHHHGSLVPRGS tag and a different multiple cloning site and in pET-20b the pelB leader sequence was replaced by an N-terminal MAHHHHHHGS tag. For the production of the cytosolic the SNARE domain, a codon-optimized synthetic gene fragment encompassing residues Gly146-Gly204 of human SNAP-25 (UniProtKB entry P60880) was cloned into the BamHI/EcoRI site of pHisTrx2, a pET-based expression vector containing an N-terminal 6xHis-tagged thioredoxin A (TrxA) fusion protein (28). Proteins were expressed in bacterial strain BL21(DE3) (NEB). Cultures were grown in LB broth at 37 °C until reaching an OD600 of 0.6, before the temperature was reduced to 18 °C. Protein expression was induced with 1 mM IPTG. Overnight expression cultures were harvested by centrifugation (4000g, 4 °C, 15 min). The proteins were purified using Ni-NTA affinity chromatography followed by size-exclusion chromatography on a Superdex 200 column. The 6xHis tag of the LC/A3 variant used for crystallization was removed by thrombin cleavage overnight at 8 °C. Cleaved samples were reapplied onto a Ni-NTA column to separate the target proteins from the tag. Pooled fractions were dialyzed in 150 mM NaCl, 20 mM Tris-HCl, pH 7.4. Protein sample purity was assessed by SDS-PAGE analysis. Protein concentration was estimated by UV at 280 nm, and proteins were aliquoted and flash frozen in liquid nitrogen and stored at −80 °C

### Proteolytic activity assay

One microgram of purified thioredoxin-SNARE fusion protein (Trx-SNAP-25) was incubated with 1 μg of purified catalytically active LC/A3 or LC/A1. Reactions were carried out in 50 μl TBS for 3 h at 25 °C or 37 °C. 10 mM ZnCl_2_ was added to the reaction mixture to check whether enzymatic activity is affected. The digested samples were analyzed on SDS-PAGE gels stained with Coomassie Blue.

### Circular dichroism (CD) spectroscopy

CD spectra of recombinant LC variants were recorded at 20 °C on a Chirascan-Plus spectrophotometer (Applied Photophysics Ltd) using a quartz cuvette of 1 mm path length. Proteins were measured using concentrations of 0.25 mg/ml (5 μM) in PBS buffer (20 mM Na_2_HPO_4_, 150 mM NaCl, pH 7.4). Spectra were recorded from 200 to 250 nm and were repeated three times. For each spectrum, the three scans were averaged and subtracted by the averaged spectrum of the PBS buffer. Thermal stability was assessed at 222 nm using a 1°C/min temperature ramp between 20 °C and 90 °C. The T_m_ for each construct was determined by fitting of the data points using the R nonlinear least square fitting function based on a sigmoid model. CDpal software was used for this purpose (29).

### LC/A3 crystallization and structure determination

LC/A3 active and inactive variants were concentrated to 8.8–15 mg/ml and crystallized by sitting-drop vapor diffusion at 20 °C. Reservoir solution (100 nl) was applied and the proteins were mixed with the mother liquor in a volume ratio of 1:1 and 2:1. Crystals of inactive truncated LC/A3 (amino acid residues Pro2-Lys417, double-mutant Glu224Gln/Phe336Tyr) were obtained in 0.2 M proline, 0.1 M HEPES 7.5 pH, and 10 %w/v PEG 3350. Crystals typically appeared within 4 days and grew to their maximum size within 1–2 weeks. A dataset to a resolution of 2 Å was collected from single, cryo-cooled crystals at beamline PXIII (Swiss Light Source, Villigen, Switzerland) equipped with an EIGER 16M high resolution diffractometer (Dectris). Raw data were processed and scaled with XDS (30). The structure was solved by molecular replacement using LC/A1-SNAP-25 structure (PDB 1XTG) as a search model (22). The structure was subsequently built and refined using PHENIX. Manual adjustments of the model were done using COOT (31). Crystallographic data and statistics are summarized in Table S1. The figures were generated with PyMOL (Schrödinger, LLC, New York)

## Data availability

Crystallographic data and coordinates were deposited in Protein Data Bank with accession number 7DVL. All remaining data are contained within the article.

## Supporting information

This article contains supporting information (20, 22, 32).

## Conflict of interest

The authors declare that they have no conflicts of interest with the contents of this article.
