# Supplementary material for: Crystal structure of the catalytic domain of botulinum neurotoxin subtype A3
Source: J Biol Chem. 2021 Apr 21;296:100684. doi: 10.1016/j.jbc.2021.100684 (PMC8135040; doi:10.1016/j.jbc.2021.100684)
Supplement: Figures and Tables [file mmc1.docx]

**Supporting information**

**Crystal structure of the catalytic domain of botulinum neurotoxin subtype A3**

Oneda Leka^†^, Yufan Wu^†^, Xiaodan Li and Richard A. Kammerer*

From the Laboratory of Biomolecular Research, Division of Biology and Chemistry, Paul Scherrer Institute, 5232 Villigen PSI, Switzerland

^1^ These authors contributed equally to this work

* To whom correspondence should be addressed: +41-56-310-4765, E-mail: richard.kammerer@psi.ch

S-1

| **LC/A1** | **LC/A3** |
| --- | --- |
| **α-exosite** | **α-Exosite** |
| **Lys356** | **Asn356** |
| **Phe357** | **Pro357** |
| Leu103 | Leu103 |
| **Ile348** | **Gly348** |
| **Lys340** | **Glu340** |
| Leu322 | Phe322 |
| Leu341 | Phe341 |
| **Lys337** | **Ala337** |
| Met344 | Val344 |
| Met106 | Met106 |
| Arg113 | Lys113 |
| Ile115 | Ile115 |
| Val316 | Ile316 |
| Thr109 | Ser109 |
| Asn40 | Glu40 |
| Tyr312 | Tyr312 |
| **Lys41** | **Gly41** |
| **Active site** | **Active site** |
| His227 | His227 |
| Thr176 | Thr176 |
| Lys166 | Lys166 |
| Phe168 | Phe168 |
| Asn136 | Asn136 |
| Pro25 | Pro25 |
| Cys134 | Cys134 |
| Val129 | Val129 |
| Trp118 | Trp119 |
| Glu148 | Glu148 |
| **β-exosite** | **β-exosite** |
| Tyr250 | Tyr250 |
| Phe369 | Phe369 |
| Leu256 | Leu256 |

**Table S1. Comparison of amino acids of LC/A1 that interact with SNAP-25 with those in LC/A3.** α-Exosite, active site and β-exosite residues that interact with SNAP-25 are shown for both LC/A1 and LC/A3. The most significant differences (in bold) are found in α-exosite α-helices 3 and 4. The order of LC/A residues is shown according to their interaction with the SNAP-25 peptide (PDB code 1XTG) (1).

S-2


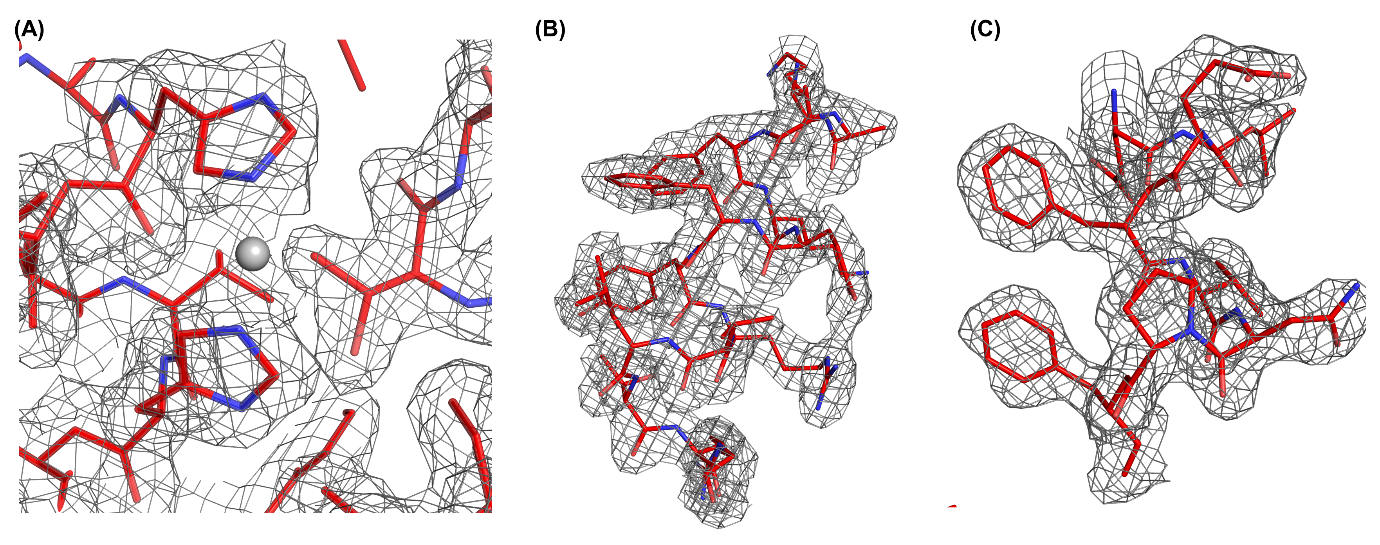


**Figure S1. Zoom-in views of refined electron density map of LC/A3 generated with the final calculated phases.** (A) Active site; (B) α-exosite α-helix 3 (residues Lys335-Ile348) and (C) α-helix 4 (residues Glu351-Phe358). The 2Fo-Fc maps were contoured at 1 σ.

S-3

**
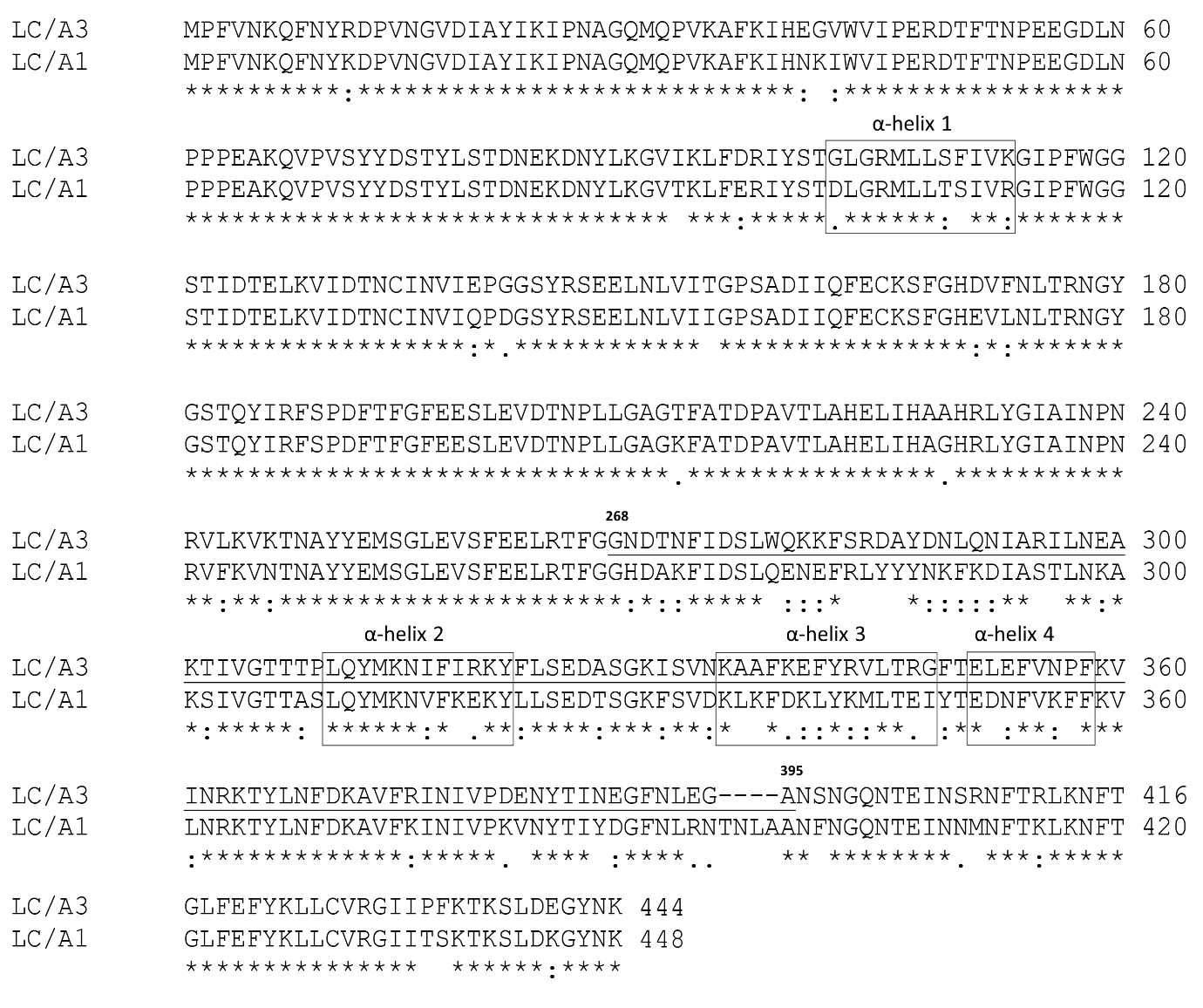
**

**Figure S2. Sequence alignment of LC/A1 (Met1-Lys448) and LC/A3 (Met1-Lys444).** The two subtypes share 81.9% sequence identity. Clustal Omega (2) was used for generating the sequence alignment. *, identical residue; :, strong similarity; ., weak similarity. The LPH region spans residues Gly268 to Ala395 of LC/A3 (underlined) (3). α-Exosite α-helices are highlighted by boxes and labelled.

S-4

**
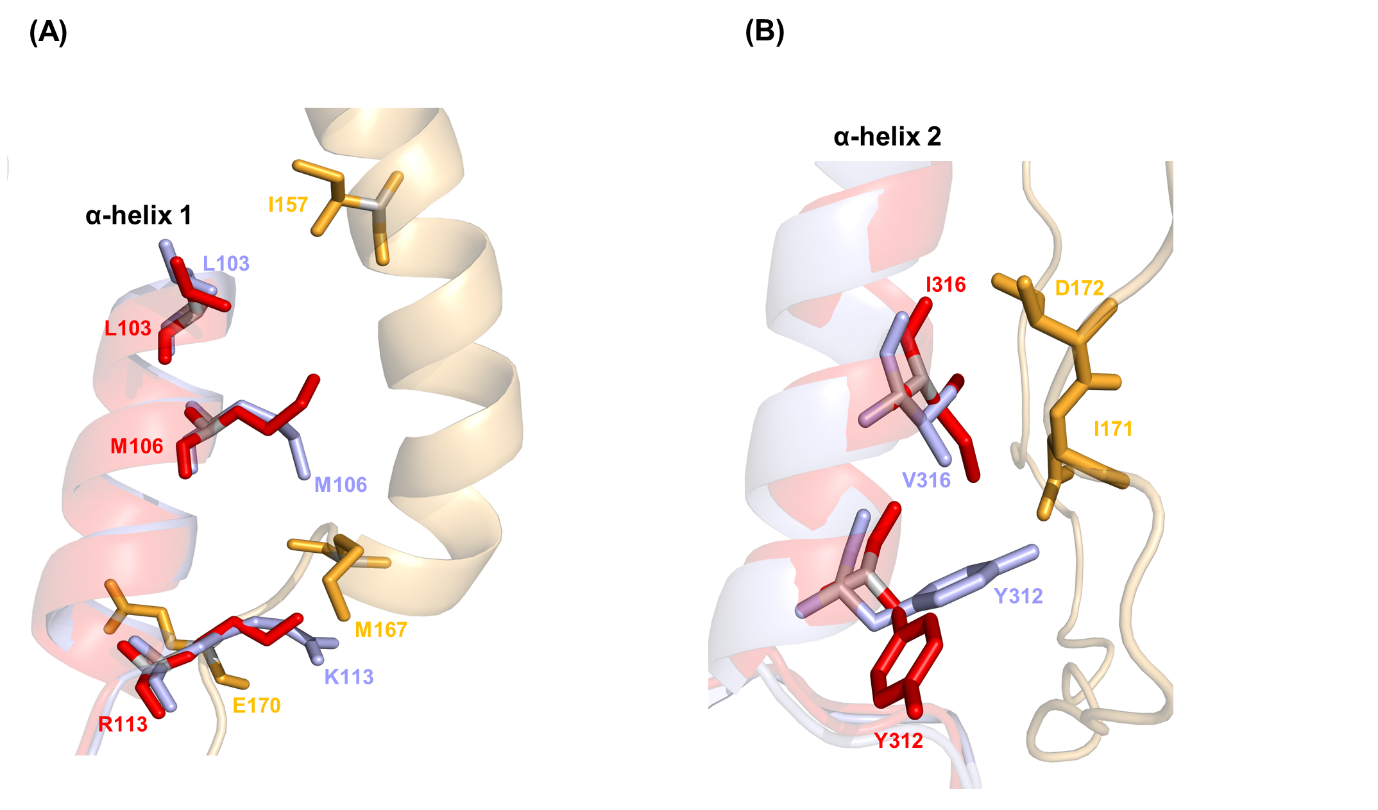
**

**Figure S3. Superimposition of ribbon representations of LC/A1 α-exosite α-helices 1 (A) and 2 (B)(magenta) interacting with SNAP-25 (gold, PDB code 1XTG) (1) and the corresponding helices of LC/A3 (red, PDB code 7DVL).** LC/A1 residues interacting with SNAP-25 amino acids and LC/A3 residues that potentially interact with the substrate are shown as sticks. α-Exosite α-helices and interacting residues are labelled.

S-5


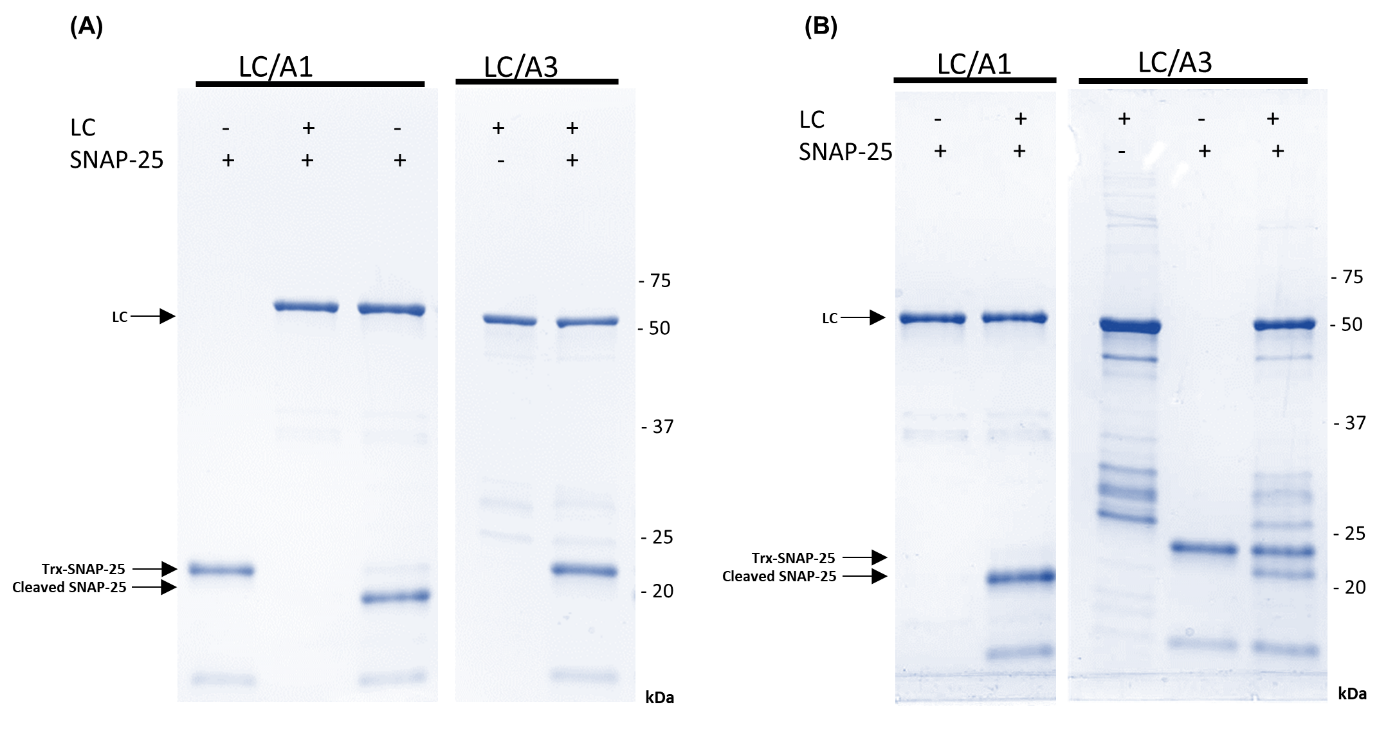


**Figure S4. Enzymatic activity of truncated LC/A1 and LC/A3.** (A) 1 µg of LC/A1 (residues Pro2-Gly421, calculated molecular mass of 49.9 kDa) or 1 µg LC/A3 (residues Pro2-Gly417, calculated molecular mass of 49.4 kDa) were incubated with 1 µg of Trx-SNAP-25 (residues Gly146-Gly204, calculated molecular mass of 20.8 kDa) in 50 µl TBS buffer for 3 hours 25 °C. (B) The same enzymatic activity assay was also performed at 37 °C. Reactions were stopped by adding SDS-PAGE buffer and heated for 5 min. Samples were subjected to 4-20% gradient SDS-PAGE and gels were stained with Coomassie Blue. LC/A1, LC/A3 and cleaved and uncleaved SNAP-25 substrate are indicated by arrows. The positions of marker proteins are indicated.

S-6


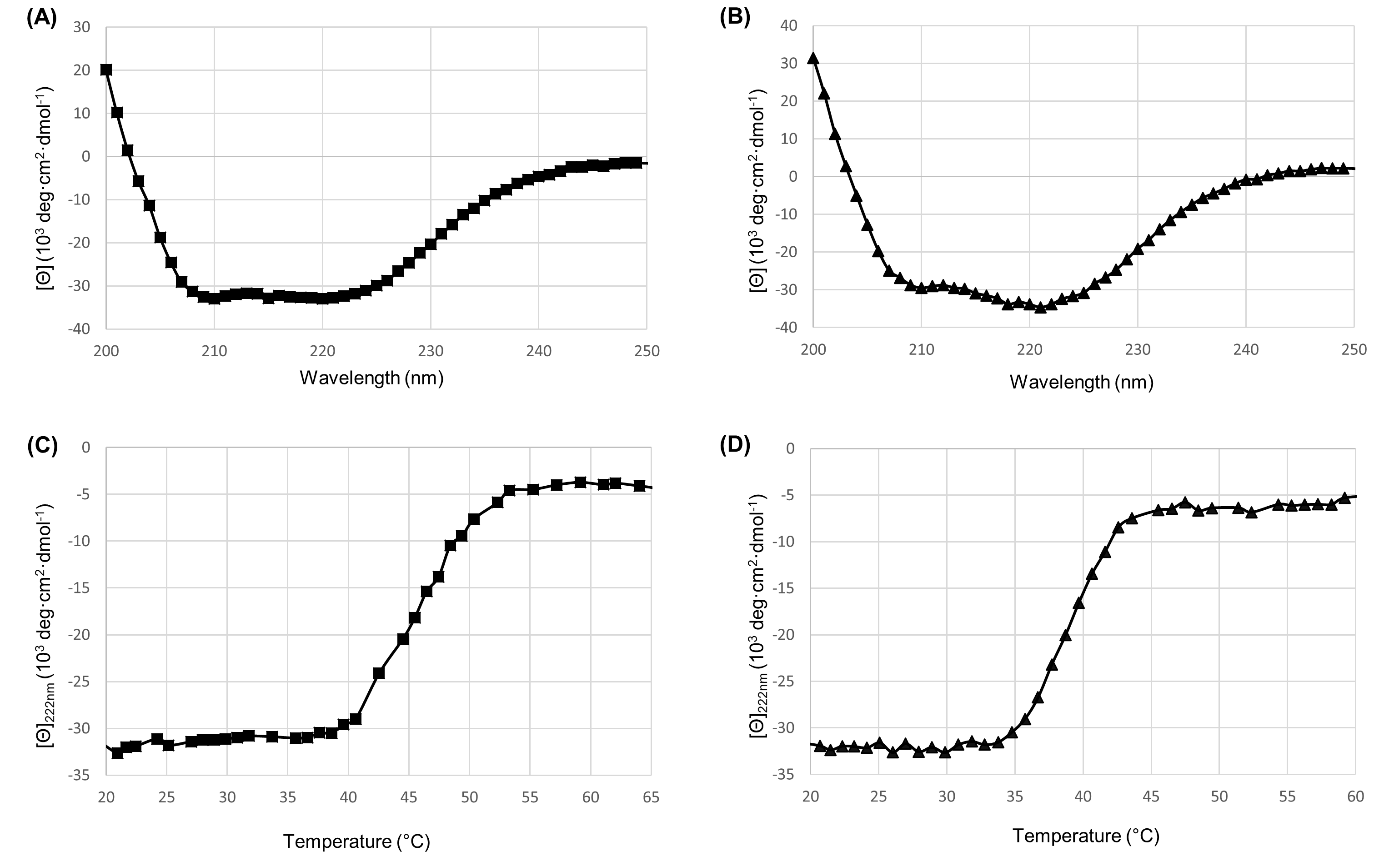


**Figure S5.** **CD spectroscopy analysis of truncated LC/A1 (residues Pro2-Gly421) and LC/A3 (residues Pro2-Gly417).** (A) and (B), CD spectra of LC/A1 and LC/A3, respectively (■, LC/A1; ▲, LC/A3). (C) and (D), thermal unfolding profiles of LC/A1 and LC/A3, respectively, recorded at 222nm. LC/A1 and LC/A3 showed concentration-independent T_m_ values of 46.5±0.2 °C and 40.1±0.1 °C, respectively, indicating that the thermal stability of LC/A3 is lower than the one of LC/A1. Proteins were measured at a concentration of 5 µM in PBS. Each spectrum is the average of three independent measurements. For the thermal unfolding profiles, a ramping rate of 1 °C/min was used.

S-7

**References**

1. Breidenbach, M. A., & Brunger, A. T. (2004). Substrate recognition strategy for botulinum neurotoxin serotype A. Nature. 432, 925–929.

2. Sievers, F., Wilm, A., Dineen, D., Gibson, T. J., Karplus, K., Li, W., Lopez, R., McWilliam, H., Remmert, M., Söding, J., Thompson, J. D., & Higgins, D. G. (2011). Fast, scalable generation of high-quality protein multiple sequence alignments using Clustal Omega. Molecular systems biology. 7, 539.

3. Pellett, S., Bradshaw, M., Tepp, W. H., Pier, C. L., Whitemarsh, R., Chen, C., Barbieri, J. T., & Johnson, E. A. (2018). The Light Chain Defines the Duration of Action of Botulinum Toxin Serotype A Subtypes. mBio. 9.

S-8
